# Are the Relationships of Lean Mass and Fat Mass With Bone Microarchitecture Causal or Due to Familial Confounders? A Novel Study of Adult Female Twin Pairs

**DOI:** 10.1002/jbm4.10386

**Published:** 2020-07-30

**Authors:** Minh Bui, Roger Zebaze, Shuai Li, John L Hopper, Åshild Bjørnerem

**Affiliations:** ^1^ Centre for Epidemiology and Biostatistics, School of Population and Global Health University of Melbourne Melbourne Victoria Australia; ^2^ Department of Medicine, School of Clinical Sciences Monash University Melbourne Victoria Australia; ^3^ Centre for Cancer Genetic Epidemiology, Department of Public Health and Primary Care University of Cambridge Cambridge UK; ^4^ Department of Clinical Medicine UiT – The Arctic University of Norway Tromsø Norway; ^5^ Department of Obstetrics and Gynecology University Hospital of North Norway Tromsø Norway

**Keywords:** BONE MICROARCHITECTURE, CAUSATION, FAT MASS, GENETIC FACTORS, LEAN MASS, TWIN PAIRS

## Abstract

It is not known whether the relationships of lean mass (LM) and fat mass (FM) with bone microarchitecture and geometry are causal and/or are because of confounders, including familial confounders arising from genetic and environment effects shared by relatives. We tested the hypotheses that: (i) LM is associated with cortical bone traits, (ii) FM is associated with trabecular bone traits, and (iii) these relationships of LM and FM with bone microarchitecture and geometry have a causal component. Total body composition was quantified for 98 monozygotic (MZ) and 54 dizygotic (DZ) white female twin pairs aged 31 to 77 years. Microarchitecture at the distal tibia and distal radius was quantified using HRpQCT and StrAx software. We applied the Inference about Causation through Examination of FAmiliaL CONfounding (ICE FALCON) method. Within‐individuals, distal tibia total bone area, cortical area, cortical thickness, and trabecular number were positively associated with LM (standardized regression coefficient (*β*) = 0.13 to 0.43; all *p* < 0.05); porosity of the inner transitional zone (ITZ) was negatively associated with LM (*β =* −0.22; *p* < 0.01). Trabecular number was positively associated with FM (*β =* 0.40; *p* < 0.001), and trabecular thickness was negatively associated with FM (*β = −*0.27; *p* < 0.001). For porosity of ITZ and trabecular number, the cross‐pair cross‐trait association with LM was significant before and after adjustment for the within‐individual association with LM (all *p*s < 0.05). For trabecular number, the cross‐pair cross‐trait association with FM was significant before and after adjustment for the within‐individual association with FM (*p* < 0.01). There were no significant changes in these cross‐pair cross‐trait associations after adjustment for the within‐individual association (*p* = 0.06 to 0.99). Similar results were found for distal radius measures. We conclude that there was no evidence that the relationships of LM and FM with bone microarchitecture and geometry are causal; they must in part due to by familial confounders affecting both bone architecture and body composition. © 2020 The Authors. *JBMR Plus* published by Wiley Periodicals LLC. on behalf of American Society for Bone and Mineral Research.

## Introduction

With advancing age, the loss of muscle (lean mass [LM]) is associated with a reduced bone mass, more falls, and increased fracture risk.^(^ ^1^, ^2^, ^3^, ^4^, ^5^, ^6^, ^7^, ^8^ ^)^ The relationship of fat mass (FM) with fracture risk is complex; low BMI is associated with low bone mass and fractures at some sites, whereas obesity is associated with fractures at other sites.^(^ ^9^, ^10^, ^11^ ^)^

When studying the relationship of bone architecture with body composition, both LM and FM must be considered independently and together.^(^ ^12^, ^13^ ^)^ When considered together, it has been found that cortical, but not trabecular, microarchitecture is associated with LM, whereas trabecular, not cortical, microarchitecture is mainly associated with FM, at least for postmenopausal women.^(^ ^12^ ^)^ Cortical porosity does not appear to be associated with LM or FM.^(^ ^12^ ^)^ Visceral adipose tissue is suggested to be a negative predictor and muscle mass a positive predictor of microarchitecture in obese men.^(^ ^14^ ^)^

A causal association of LM on bone microarchitecture and geometry has been proposed.^(^ ^12^, ^15^ ^)^ The mechanostatic hypothesis proposes that muscle contractions apply forces to bones that cause deformations or strains within the bone tissue.^(^ ^15^ ^)^ These forces are sensed by osteocytes, which increase bone formation through the bone‐remodeling process, resulting in increased cortical area and thickness, and increased bone strength. It has been argued that the associations of bone size with both muscle size (forearm and lower leg cross‐sectional area of muscle) and grip strength support the mechanostat hypothesis and a role of the muscle–bone unit.^(^ ^4^, ^12^ ^)^ However, as the association of muscle size with bone structure is stronger than the association of muscle strength, other mechanisms could be involved, such as genetic, developmental, or hormonal factors.^(^ ^4^, ^12^ ^)^ Dietary factors and physical activity have also been proposed to play a role in explaining the association between bone traits and muscle.^(^ ^1^, ^7^, ^12^ ^)^

A causal association of FM with bone traits has been proposed through the action of estrogen.^(^ ^12^ ^)^ A beneficial association of estrogen on bone is well‐established,^(^ ^16^, ^17^, ^18^ ^)^ as shown by bone loss after the drop in serum estrogen levels across the menopausal transition.^(^ ^19^, ^20^ ^)^ Estrogen produced by adipocytes is an important source of estrogen for postmenopasual women.^(^ ^21^ ^)^

Twin studies have predicted that 42%–92% of the variance in bone mass,^(^ ^1^, ^22^, ^23^ ^)^ 50% to 80% of the variance in bone microarchitecture,^(^ ^24^ ^)^ and 52% to 84% of the variance in LM,^(^ ^2^, ^22^ ^)^ and 65% of the variance in LM^(^ ^22^ ^)^ are due to genetic factors. During the last decade, genome‐wide association studies have identified single‐nucleotide polymorphisms in many loci associated with BMD,^(^ ^25^, ^26^, ^27^ ^)^ cortical porosity and trabecular bone fraction,^(^ ^28^ ^)^ LM^(^ ^29^ ^)^ and BMI in adults,^(^ ^30^ ^)^ Mendelian randomization uses genetic variants to infer whether risk factors have a causal influence on health outcomes under strong assumptions, and found evidence that adiposity‐related traits have a causal effect on BMD at the heel for children.^(^ ^31^ ^)^ In addition, deletion of genome‐wide association study‐identified genes has been shown to result in increased cortical porosity and decreased bone strength of KO mice.^(^ ^27^ ^)^ We had previously investigated if genetic factors explained the associations between LM and bone density using a twin study.^(^ ^1^ ^)^ In that study we assumed that the LM measures did not have a causal effect on bone density.

To the best of our knowledge, it is not known whether the relationships of LM and FM with bone microarchitecture are causal, and/or due to genetic or environmental confounders. We therefore tested the hypotheses that: (i) LM is associated with cortical bone traits, (ii) FM is associated with trabecular bone traits, and (iii) these relationships of LM and FM with bone microarchitecture and other bone traits are causal. We did this applying a novel approach to the analysis of a twin study, Causation through Examination of FAmiliaL CONfounding (ICE FALCON), which allows inference on causation to be made from examination of familial confounding and changes in pairs of regression coefficients.^(^ ^32^ ^)^

## Materials and Methods

### Study sample

This twin study included 324 female twin pairs, 199 monozygotic (MZ) and 125 dizygotic (DZ), aged 31 to 77 years at baseline and was conducted in Melbourne, Australia from 2008 to 2011.^(^ ^19^, ^24^, ^32^, ^33^, ^34^ ^)^ At follow‐up in 2011 to 2013, participants had a total body scan for assessment of body composition. After excluding 39 women who had been treated with hormone replacement therapy or whose bone scans had movement artifacts, 388 women had valid measurements of distal tibia at the follow‐up visit. Of these, we excluded 24 women with missing total body scans and 60 for whom we did not have measurements for their cotwin. This left 152 complete pairs (54 DZ and 98 MZ) for the analysis of distal tibia traits. After a similar exclusion process, there were 124 complete pairs (45 DZ and 79 MZ) for the analysis of distal radius traits. All women had given written informed consent. The study was approved by the Austin Health Human Research Ethics Committee.

### Bone microstructure and other measurements

Three‐dimensional HRpQCT (isotropic resolution of 82 μm; XtremeCT; Scanco Medical AG, Brüttisellen, Switzerland) was used to obtain images at 60 kVp using 900 μA at the nondominant distal tibia and distal radius.^(^ ^35^, ^36^ ^)^ The region of interest consisted of 110 CT slices obtained at 22.5 and 9.5 mm from a reference line at the endplate of the distal tibia and distal radius, respectively. The 49 most proximal slices were chosen because the relatively thicker cortex allows accurate assessment of porosity. Porosity within the total cortex and its compartments (compact cortex, outer and inner transitional zones [TZs]), matrix mineralization density, trabecular number, thickness, separation, and total, cortical, and trabecular cross‐sectional area (CSA), total, cortical, and trabecular volumetric bone mineral density (vBMD) were quantified using StrAx software (Straxcorp, Melbourne, Australia), a nonthreshold‐based method that automatically segments bone from background and into its compartments. The precision was 0.5% to 3.0%.^(^ ^37^, ^38^ ^)^ Daily quality control was carried out by scanning a phantom containing rods of hydroxyapatite (QRM, Moehrendorf, Germany). Cortical and trabecular microstructure was derived based on the photon attenuation by mineralized bone. Porosity is the proportion of voxels within the cortical compartment that contains void. Once deposited, osteoid is mineralized reaching ≥80% of full mineralization (1,200 mg HA/cm^3^) within days. Matrix mineralization is quantified as the mean density of voxels with attenuation between 80% to 100% of fully mineralized bone. These voxels are unlikely to contain a pore because a pore results in voxel attenuation <80% of the maximum. So, variation in attenuation within 80% to 100% of full mineralization reflects heterogeneity in mineralization. Voxels with attenuating <80% are used to calculate porosity. Total body LM and FM were quantified using DXA (Lunar, Madison, WI, USA).

### Statistical methods

Summary statistics were presented as mean and SD. Within‐pair correlations were estimated for MZ and DZ pairs, and Fisher's *z*‐transform was used to test for differences in correlations between these two groups. To test whether the MZ twins bone traits were similar to the DZ twins, we compared trait means between these two groups, adjusted for age and height, using the generalized estimating equation method, which takes into account correlation within twin pairs.

This method was also used to apply the Inference about Causation through Examination of FAmiliaL CONfounding (ICE FALCON) models so as to investigate whether there was evidence consistent with an association being due to causal effects or to familial confounding.^(^ ^32^, ^39^, ^40^ ^)^

In brief, three models were fitted to the data in regression analysis using the generalized estimating equation method to allow for the outcomes being correlated within twin pairs. The first model estimated within individual‐association of LM or FM with each bone trait, giving β_*self*_. The second model estimated the cross‐pair cross‐trait association between LM or FM for one twin and a bone trait for the other twin, giving β_*cotwin*_. The third model estimated the within‐individual and cross‐pair cross‐trait associations concurrently, in effect adjusting each predictor for the other, giving $\beta_{\text{self}}^{\mathrm{adj}}$ and $\beta_{\text{cotwin}}^{\mathrm{adj}}$. If a predictor has a causal effect on the outcome, the cross‐pair cross‐trait association will be attenuated towards zero (β_*cotwin*_ > $\beta_{\text{cotwin}}^{\mathrm{adj}}$), but the within‐individual association will be unchanged (β_*self*_ = $\beta_{\text{self}}^{\mathrm{adj}}$). Hypothesis testing of the changes in regression coefficients from before and after adjustment, β_*self*_ − $\beta_{\text{self}}^{\mathrm{adj}}$ and β_*cotwin*_ − $\beta_{\text{cotwin}}^{\mathrm{adj}}$, was conducted by using the method proposed by Yan and colleagues,^(^ ^41^ ^)^ implemented in the R package “geepack,”^(^ ^42^ ^)^ to estimate the standard errors of the changes. Because of small sample size for DZ twins (54 pairs), which does not have power to detect significant cross‐pair cross‐trait association, we therefore conducted ICE FALCON analyses for the combined MZ and DZ twin pairs. All models were adjusted for age and height. Outcome and predictor variables were all standardized to have a mean of zero and SD of 1; all *p* values were two‐sided. Furthermore, we also conducted simulation studies to assess the performance of the changes in coefficients under causal effect and familial confounding (see Supplemental Information). The ICE FALCON and all other analyses were conducted using our own programs written in R language (R Foundation for Statistical Computing, Vienna, Austria; https://www.r-project.org/). Following convention, we have defined statistical significance as *p* < 0.05 and not adjusted for multiple comparisons.

## Results

The mean (SD) of age was 50.8 years (8.1). The MZ and DZ twins did not differ in mean age, LM, FM, and distal tibial bone traits, but MZ twins were shorter than DZ twins (161.9 versus 164.1 cm; *p* = 0.019; Table 1). Within‐pair correlations, adjusted for age and height, were higher for MZ pairs [*r*(MZ)] than for DZ pairs [*r*(DZ)] for LM (0.79 versus 0.54; *p* = 0.003) and for FM (0.79 versus 0.40, *p* < 0.001).

**Table 1:** Characteristics of and Comparison Between Dizygotic (DZ) and Monozygotic (MZ) Twins

	DZ (*n* = 108)		MZ (*n* = 196)		*p*
	Mean	SD	Mean	SD	
Age (years)	50.3	6.26	51.0	8.95	0.585
Height (cm)	164.1	6.18	161.9	5.77	0.019
Weight (kg)	71.0	15.0	69.1	14.9	0.413
Total body lean mass (kg)	40.2	4.66	39.7	5.11	0.544
Total body fat mass (kg)	27.4	11.2	26.6	11.1	0.646
Distal tibia bone traits					
Total bone CSA (mm^2^)	632	104	608	93.8	0.116
Total vBMD (mg HA/cm^3^)	307	59.6	311	52.8	0.596
Total BMC (mg HA)	763	126	750	119	0.482
Cortical CSA (mm^2^)	210	20.9	207	21.0	0.432
Cortical CSA/Total CSA	33.8	4.86	34.6	4.58	0.271
Cortical thickness (mm)	2.40	0.25	2.43	0.25	0.516
Total cortex porosity (%)	62.2	6.29	61.2	5.24	0.241
Compact cortex porosity (%)	43.9	7.61	42.7	6.26	0.295
Outer TZ porosity (%)	44.8	6.92	43.8	5.44	0.312
Inner TZ porosity (%)	85.6	3.14	85.8	2.54	0.775
Cortical vBMD (mg HA/cm^3^)	640	80.5	653	66.4	0.253
Cortical BMC (mg HA)	538	82.8	544	79.3	0.644
Matrix mineralization density (%)	64.2	1.49	64.4	1.30	0.489
Medullary CSA (mm^2^)	422	95.3	401	84.5	0.122
Medullary CSA/Total CSA	66.2	4.86	65.4	4.58	0.271
Trabecular number (1/mm)	2.32	0.52	2.29	0.50	0.688
Trabecular thickness (mm)	0.20	0.01	0.20	0.01	0.822
Trabecular separation (mm)	1.44	0.28	1.50	0.28	0.195
Trabecular vBMD (mg HA/cm^3^)	133	37.8	128	36.2	0.345
Trabecular BMC (mg HA)	225	83.0	206	76.0	0.114

CSA = Cross‐sectional area; HA = hydroxyapatite; TZ = transitional zone; vBMD = volumetric bone mineral density.

Univariably, greater LM was associated with greater total bone CSA (standardized regression coefficient β = 0.13; *p* < 0.05), and both greater LM and greater FM were associated higher total BMC (β = 0.36; *p* < 0.01 and β = 0.317; *p* < 0.001, respectively), larger cortical CSA, thicker cortices and lower cortical porosity, and increased trabecular number of thinner trabeculae (absolute β ranges from 0.11 to 0.43; all *p*s < 0.05; see Table 2). When fitted together, greater LM was associated with larger total bone CSA, higher total BMC, larger cortical CSA, thicker cortices, lower porosity of the inner TZ, and increased trabecular number (absolute β ranges from 0.18 to 0.47; all *p*s < 0.05), but the strength of association for the latter was attenuated. Greater FM was no longer associated with the cortical bone traits after adjustment for LM, but remained associated with increased trabecular number and thinner trabeculae (absolute β ranges from 0.22 to 0.34; all *p*s < 0.001).

**Table 2:** Within‐Individual Associations (Regression Coefficient b and Standard Error) of Lean Mass and Fat Mass (Predictors) With Distal Tibia Bone Traits Adjusted for Age and Height (Outcomes)

Distal tibia bone traits	Univariable models		Mutually adjusted models	
	Lean mass (kg)	Fat mass (kg)	Lean mass (kg)	Fat mass (kg)
	b ± SE	b ± SE	b ± SE	b ± SE
Total bone CSA (mm^2^)	0.134 ± 0.068*	−0.002 ± 0.048	0.181 ± 0.069**	−0.071 ± 0.154
Total vBMD (mg HA/cm^3^)	0.184 ± 0.062**	0.133 ± 0.043**	0.127 ± 0.071	0.086 ± 0.050
Total BMC (mg HA)	0.364 ± 0.069**	0.167 ± 0.043***	0.332 ± 0.074***	0.048 ± 0.049
Cortical CSA (mm^2^)	0.425 ± 0.073***	0.110 ± 0.050*	0.473 ± 0.081***	−0.071 ± 0.061
Cortical CSA/Total CSA	0.131 ± 0.064*	0.071 ± 0.049	0.112 ± 0.067	0.028 ± 0.053
Cortical thickness (%)	0.322 ± 0.074***	0.112 ± 0.054*	0.331 ± 0.078***	−0.014 ± 0.060
Total cortex porosity (%)	−0.146 ± 0.059*	−0.110 ± 0.035**	−0.097 ± 0.066	−0.074 ± 0.040
Compact cortex porosity (%)	−0.033 ± 0.058	−0.006 ± 0.039	−0.039 ± 0.061	0.009 ± 0.040
Outer TZ porosity (%)	−0.027 ± 0.055	0.011 ± 0.029	−0.045 ± 0.064	0.028 ± 0.034
Inner TZ porosity (%)	−0.215 ± 0.072**	−0.098 ± 0.051	−0.200 ± 0.086*	−0.022 ± 0.063
Cortical vBMD (mg HA/cm^3^)	0.145 ± 0.059*	0.112 ± 0.035**	0.094 ± 0.067	0.077 ± 0.040
Cortical BMC (mg HA)	0.398 ± 0.069***	0.161 ± 0.044***	0.387 ± 0.074***	0.017 ± 0.051
Matrix mineralization density (%)	0.040 ± 0.057	0.004 ± 0.035	0.050 ± 0.066	−0.015 ± 0.041
Medullary CSA (mm^2^)	0.050 ± 0.068	−0.027 ± 0.049	0.091 ± 0.068	−0.061 ± 0.050
Medullary CSA/Total CSA	−0.131 ± 0.064*	−0.071 ± 0.049	−0.112 ± 0.067	−0.028 ± 0.053
Trabecular number (1/mm)	0.390 ± 0.067***	0.401 ± 0.055***	0.177 ± 0.066**	0.335 ± 0.060***
Trabecular thickness (mm)	−0.243 ± 0.071***	−0.272 ± 0.047***	−0.082 ± 0.078	−0.240 ± 0.054***
Trabecular separation (mm)	−0.247 ± 0.062***	−0.262 ± 0.050***	−0.103 ± 0.070	−0.224 ± 0.056***
Trabecular vBMD (mg HA/cm^3^)	0.149 ± 0.070*	0.121 ± 0.050*	0.093 ± 0.082	0.087 ± 0.058
Trabecular BMC (mg HA)	0.134 ± 0.068	0.087 ± 0.050	0.102 ± 0.071	0.050 ± 0.051

CSA = cross‐sectional area; HA = hydroxyapatite; TZ = transitional zone; vBMD = volumetric bone mineral density.

Outcome variables and predictors were standardised to have mean zero and standard deviation of 1.

* *p* < 0.05.

** *p* < 0.01.

*** *p* < 0.001.

We conducted ICE FALCON analyses of the bone traits association with LM or FM based on the significant associations found from the analyses in Table 2 (see Table 3). The cross‐pair cross‐trait association of LM with the distal tibia total BMC and cortical BMC, porosity of the inner TZ, and trabecular number remained significant after adjustment for the respective within‐individual association. The cross‐pair cross‐trait association of FM with distal tibia trabecular number, separation, and vBMD remained after adjustment for the respective within‐individual association. None of the changes in the cross‐pair cross‐trait associations, i.e., β_*cotwin*_ – $\beta_{\text{cotwin}}^{\mathrm{adj}}$, was significant (*p* ranging from 0.149 to 0.998), except marginally for cortical CSA with LM (*p* = 0.062), as well as the changes in the within‐individual association, β_*self*_ – $\beta_{\text{self}}^{\mathrm{adj}}\;$(all *p*s > 0.100, results not shown). The results in Table 3 suggest that the associations between bone trait and LM and FM were confounded by familial factors, and this was confirmed by simulation results (Supplementary Table S1), where estimated biases and mean square errors were smaller for the model simulated under familial confounding than for the model in which LM or FM causes the bone trait (again except for cortical CSA). For the distal radius, the associations were weaker, but they followed similar patterns as for the distal tibia (Supplementary Table S2), and because there was no significant cross‐pair cross‐trait association, no further analysis was performed (Supplementary Table S3).

**Table 3:** ICE FALCON Analyses for the Associations (Regression Coefficients b and Standard Error) of Distal Tibia Bone Traits Adjusted for Age and Height (Outcomes) With Lean Mass and Fat Mass (Predictors)

	Univariable cross‐pair cross‐trait association			Cross‐pair cross‐trait association allowing for within‐individual association			Absolute change in cross‐pair cross‐trait association	
Distal tibia bone traits (outcomes)	β_cotwin_	SE	*p*	$\beta_{\text{cotwin}}^{\mathrm{adj}}$	SE	*p*	Change	*p*
Lean mass (predictor)								
Total bone CSA (mm^2^)	0.028	0.053	0.609	0.018	0.056	0.741	−0.009	0.494
Total BMC (mg HA)	0.183	0.055	0.001	0.143	0.050	0.004	−0.040	0.220
Cortical CSA (mm^2^)	0.150	0.068	0.028	0.072	0.059	0.223	−0.079	0.062
Cortical thickness (mm)	0.113	0.070	0.110	0.078	0.061	0.197	−0.034	0.249
Inner TZ porosity (%)	−0.150	0.059	0.011	−0.122	0.058	0.035	0.028	0.169
Cortical BMC (mg HA)	0.144	0.061	0.018	0.108	0.049	0.028	−0.036	0.312
Trabecular number (1/mm)	0.159	0.057	0.006	0.112	0.050	0.024	−0.047	0.149
Fat mass (predictor)								
Trabecular number (1/mm)	0.162	0.052	0.002	0.162	0.041	0.001	0.0001	0.998
Trabecular separation (mm)	−0.133	0.045	0.003	−0.150	0.044	0.001	−0.017	0.533
Trabecular vBMD (mg HA/cm^3^)	0.137	0.042	0.001	0.152	0.045	0.001	0.015	0.298

Outcome and predictor variables were standardized to have mean of zero and SD of 1, *p* were for two‐sided.

CSA = Cross‐sectional area; HA = hydroxyapatite; ICE FALCON = Inference about Causation through Examination of FAmiliaL CONfounding; TZ = transitional zone; vBMD = volumetric bone mineral density.

## Discussion

For adult women, we found that within an individual the cortical bone traits were mainly associated with LM, not FM, and the trabecular bone traits were mainly associated with FM. For each of these body compositions—bone trait associations—we found that the body composition trait of a twin was associated with the bone trait of the cotwin. But these cross‐pair cross‐trait associations did not change after adjustment for the individual's body composition. Following the logic of the ICE FALCON approach, we have found no evidence that the body composition measure of an individual had a causal effect on their bone trait, except for cortical CSA and LM. Given the cross‐pair cross‐trait associations with LM and FM were significant after adjustment for the respective within‐individual associations with LM and FM, the cross‐pair cross‐trait associations must be attributable, at least in part, to familial factors shared by twins in the same pair, and these could include both genetic and environmental factors.

We confirmed that greater LM was associated with a larger bone size and cortical area, as well as thicker cortices independent of FM.^(^ ^12^ ^)^ In addition, greater LM was associated with a lower cortical porosity of the inner transitional zone—a novel finding. Measurement of porosity in the transitional zone was made possible by an accurate segmentation of bone using StrAx software. As previously reported, bone loss due to unbalanced remodeling upon intracortical canal surfaces starts and is more pronounced in the portion of the cortex adjacent to the marrow space, which corresponds to the inner transitional zone.^(^ ^43^ ^)^ This might explain why the greater LM was associated with lower cortical porosity of the inner transitional zone only. We also confirmed that greater FM was associated with an increased trabecular number,^(^ ^12^ ^)^ but we also found that greater LM was associated with that outcome even after adjusting for FM. Having a larger sample size of 304 women in the current study versus 167 women in the previous study^(^ ^12^ ^)^ could be one reason why we found that both FM and LM are associated with a higher trabecular number.

The developmental origins hypothesis proposes that the body composition and bone traits may be correlated because both are associated with early life environment.^(^ ^12^ ^)^ However, twin studies have found that the variances in bone mass, LM, and FM appear to be largely determined by genetic factors.^(^ ^1^, ^2^, ^22^, ^23^ ^)^ The heritabilities for the cortical and trabecular traits were estimated to be from 67% to 88% based on the twin baseline data, under the assumptions of the classic twin model.^(^ ^24^ ^)^ Here we have found evidence consistent with those high heritabilities for LM and FM under the same assumptions, in keeping with previous reports.^(^ ^2^, ^22^ ^)^ Whether the relationships of bone traits with LM or FM also are determined solely by genetic factors is not known.^(^ ^1^ ^)^

This study has several limitations. We cannot exclude causal roles of LM and FM as they might not be detectable with our sample size. Larger sample sizes are needed to examine the hypotheses with more power and to provide more precision on the amount of cross‐pair cross‐trait correlations that could be explained by a causal relationship of bone geometry and microarchitecture with LM and FM. Our approach is also novel, so replication studies using a twin or sister‐pair design for the prospective evaluation of the relationships of changes in bone traits with LM and FM will be important in trying to validate our findings and approach. The clinical utility is to not delude adult women of this age into thinking there is evidence that changing their FM or LM will have a causal effect on the bone measures.

In conclusion, noninvasive assessment of bone morphology is feasible and increasingly available. Use of this methodology to study the skeletons of twin pairs permits insights into the pathophysiology of bone fragility. Here, we report that greater LM was associated with larger bone size and improved cortical and trabecular microarchitecture, and FM was associated with improved trabecular microarchitecture, but these associations were not causal for adult women, or not strong enough to be detectable by this study. This issue needs to be addressed separately for younger women, including children with growing bones, because any evidence for causation would have important implications for prevention of fracture. Given the familial nature of the cross‐pair cross‐trait associations, there must be familial factors that predispose to both the body composition and the bone traits. These familial factors could have genetic or environmental origins, or both.

## Disclosures

RZ is director and shareholder of StrAx Corp Pty Ltd and has received research grants, served on the advisory boards, and/or received honoraria from Amgen, MSD, Servier, and Sanofi Genzyme. All authors state that they have no other conflicts of interest.

## Peer Review

The peer review history for this article is available at https://publons.com/publon/10.1002/jbm4.10386.

## Supporting information


**Appendix S1** Supplementary material.
**Supplementary Table S1** Simulation results for the relationships examined in the Table 3 where cross‐pair cross‐trait associations were significant
**Supplementary Table S2** Within‐individual associations (regression coefficients b and standard error (s.e.)) of lean mass and fat mass (predictors) with distal radius bone traits adjusted for age and height (outcomes).
**Supplementary Table S3** ICE FALCON analyses for the associations of distal radius bone traits adjusted for age and height (outcomes) with lean mass and fat mass (predictors)
