# Supplementary material for: Are the Relationships of Lean Mass and Fat Mass With Bone Microarchitecture Causal or Due to Familial Confounders? A Novel Study of Adult Female Twin Pairs
Source: JBMR Plus. 2020 Jul 30;4(9):e10386. doi: 10.1002/jbm4.10386 (PMC7507375; doi:10.1002/jbm4.10386)
Supplement: Supplementary file 1 — Appendix S1 Supplementary material. Supplementary Table S1 Simulation results for the relationships examined in the Table 3 where cross‐pair cross‐trait associations were significant Supplementary Table S2 Within‐individual associations (regression coefficients b and standard error (s.e.)) of lean mass and fat mass (predictors) with distal radius bone traits adjusted for age and height (outcomes). Supplementary Table S3 ICE FALCON analyses for the associations of distal radius bone traits adjusted for age and height (outcomes) with lean mass and fat mass (predictors) [file JBM4-4-e10386-s001.pdf]

Supplemental data (simulation)

To test performance of ICE FALCON inference we conducted simulation study of the two possible causal scenarios: association due to familial confounding and lean mass or fat mass causes bone trait. The simulation was conducted based on correlations of the observed data, namely within-twin correlation for each trait and within-individual cross trait correlation, and carried out as follow. Let R_X be the residual of the predictor X after regresses on the confounders, and similarly defined R_Y for outcome Y on the same confounders, ρ_X be the within twin pairs correlation for R_X, ρ_Y for R_Y and ρ_{XY} for cross-trait correlation between R_X and R_Y. Defined $\overline{R}_X$ and SD_{R_X} be the mean and standard deviation for R_X and similarly $\overline{R}_Y$ and SD_{R_Y} for R_Y. The simulated data was generated with the sample size equal to that of the original data and simulated according to the following scenarios.

For Review Only

1. X causes Y. Generate uncorrelated standard normal random variables $Z_1, \ldots, Z_6$ with the sample size (N) equals to that of the number of twin pairs in the data, then compute simulated X as follow,

$$X_{i1}^{sim} = X_{i1}^{*} SD_{R_X} + \overline{R}_X \quad \text{and} \quad X_{i2}^{sim} = X_{i2}^{*} SD_{R_X} + \overline{R}_X$$

where $i = 1, \ldots, N$,

$$X_{i1}^{*} = Z_1\sqrt{\rho_X} + Z_2\sqrt{1-\rho_X} \quad \text{and } X_{i2}^{*} = Z_1\sqrt{\rho_X} + Z_3\sqrt{1-\rho_X}\ .$$

The simulated Y was carried out as follow,

$$Y_{i1}^{sim} = Y_{i1}^{*} SD_{R_Y} + \overline{R}_Y \quad \text{and} \quad Y_{i2}^{sim} = Y_{i2}^{*} SD_{R_Y} + \overline{R}_Y$$

where

$$Y_{i1}^{*} = \rho_{XY}X_{i1}^{*} + Z_4\sqrt{\rho_Y - \rho_{XY}^2\rho_X} + Z_5\sqrt{1-\rho_{XY}^2 - (\rho_Y - \rho_{XY}^2\rho_X)}$$

and

$$Y_{i2}^{*} = \rho_{XY}X_{i2}^{*} + Z_4\sqrt{\rho_Y - \rho_{XY}^2\rho_X} + Z_6\sqrt{1-\rho_{XY}^2 - (\rho_Y - \rho_{XY}^2\rho_X)}\ .$$

2. Familial Confounding. For the case of familial confounding, generate an extra uncorrelated normal random variable Z_7, then X_{ij}^{*} and Y_{ij}^{*} (j =1, 2) are generated as follow:

$$X_{i1}^{*} = Z_1\sqrt{\rho_X - \rho_{XY}} + Z_2\sqrt{\rho_{XY}} + Z_3\sqrt{1-\rho_X}\ ,$$

$$X_{i2}^{*} = Z_1\sqrt{\rho_X - \rho_{XY}} + Z_2\sqrt{\rho_{XY}} + Z_4\sqrt{1-\rho_X},$$

$$Y_{i1}^{*} = Z_5\sqrt{\rho_Y - \rho_{XY}} + Z_2\sqrt{\rho_{XY}} + Z_6\sqrt{1 - \rho_X}\,,$$

and

$$Y_{i2}^{*} = Z_5\sqrt{\rho_Y - \rho_{XY}} + Z_2\sqrt{\rho_{XY}} + Z_7\sqrt{1 - \rho_X}.$$

The values for X_{ij}^{sim} and Y_{ij}^{sim} (j =1, 2) are then computed as in 1.

Let S be the number of simulations (i.e s = 1, 2, 3,…..S), $\hat{\beta}$ be the estimated parameter obtained from the ICE FALCON models from the actual data (i..e $\hat{\beta}_{cotwin}$- $\hat{\beta}_{cotwin}^{adj}$) and $\hat{\theta}_s$ be the estimated parameter obtained from the i*th* simulation. We used the following statistics (bias and mean square error (MSE)) to assess the performance of ICE FALCON models under the two scenarios:

$$\widehat{Mean} = \frac{1}{S}\sum_{s=1}^{S}\hat{\theta}_s = \bar{\theta}$$

$$\widehat{Bias} = \bar{\theta} - \hat{\beta}$$

$$\widehat{MSE} = \frac{1}{S}\sum_{s=1}^{S}(\hat{\theta}_s - \hat{\beta})^2$$

A scenario with smaller estimated bias and MSE gave a better performance.

For Review Only

Table S1: Simulation results for the relationships examined in the Table 3 where cross-pair cross-trait associations were significant.

Outcome	Causal Scenario	*Bias* ×1000	*MSE* ×1000
Lean Mass (predictor)			
Total BMC	Familial confounding	5.28	2.52
	X causes Y	42.7	7.23
Cortical CSA	Familial confounding	19.2	16.5
	X causes Y	8.84	3.95
Inner TZ porosity	Familial confounding	3.67	0.49
	X causes Y	7.58	1.22
Cortical BMC	Familial confounding	2.80	1.12
	X causes Y	41.9	6.25
Trabecular number	Familial confounding	6.45	1.26
	X causes Y	24.7	4.90
Fat Mass (predictor)			
Trabecular Number	Familial confounding	33.1	2.64
	X causes Y	162	32.0
Trabecular Separation	Familial confounding	8.99	0.77
	X causes Y	55.7	5.27
Trabecular vBMD	Familial confounding	0.59	0.28
	X causes Y	22.9	1.30

MSE = Mean square errors; X = lean mass or fat mass; Y = bone traits. Bias and MSE were computed for $\hat{\beta}_{cotwin}$- $\hat{\beta}^{adj}_{cotwin}$.

For Review Only

Table S2. Within-individual associations (regression coefficients b and standard error (s.e.)) of lean mass and fat mass (predictors) with distal radius bone traits adjusted for age and height (outcomes)

**Distal radius bone traits**	Univariable models		Mutually adjusted models	
	Lean mass	Fat mass	Lean mass	Fat mass
	b ± s.e.	b ± s.e.	b ± s.e.	b ± s.e.
Total bone CSA (mm²)	0.104 ± 0.072	0.003 ± 0.056	0.140 ± 0.077	-0.055 ± 0.064
Total vBMD (mg HA/cm³)	0.123 ± 0.082	0.140 ± 0.057*	0.040 ± 0.083	0.123 ± 0.059*
Total BMC (mg HA)	0.270 ± 0.071***	0.185 ± 0.052***	0.199 ± 0.081*	0.103 ± 0.061
Cortical CSA (mm²)	0.244 ± 0.059***	0.118 ± 0.054*	0.228 ± 0.078**	0.024 ± 0.070
Cortical CSA/Total CSA	0.084 ± 0.088	0.105 ± 0.062	0.019 ± 0.081	0.098 ± 0.057
Cortical thickness (mm)	0.224 ± 0.105*	0.189 ± 0.080*	0.136 ± 0.096	0.132 ± 0.073
Total cortex porosity (%)	-0.068 ± 0.076	-0.110 ± 0.054*	0.010 ± 0.080	-0.115 ± 0.059
Compact cortex porosity (%)	0.009 ± 0.062	0.003 ± 0.049	0.010 ± 0.064	-0.002 ± 0.051
Outer TZ porosity (%)	-0.056 ± 0.059	-0.014 ± 0.046	-0.065 ± 0.061	0.012 ± 0.047
Inner TZ porosity (%)	-0.144 ± 0.080	-0.109 ± 0.058	-0.097 ± 0.089	-0.069 ± 0.063
Cortical vBMD (mg HA/cm³)	0.072 ± 0.076	0.113 ± 0.054*	-0.007 ± 0.081	0.116 ± 0.059*
Cortical BMC (mg HA)	0.230 ± 0.074**	0.173 ± 0.053**	0.155 ± 0.081	0.110 ± 0.059
Matrix mineralization density (%)	0.012 ± 0.069	-0.028 ± 0.051	0.042 ± 0.082	-0.046 ± 0.060
Medullary CSA (mm²)	0.043 ± 0.081	-0.036 ± 0.059	0.092 ± 0.080	-0.074 ± 0.061
Medullary CSA/Total CSA	-0.084 ± 0.088	-0.105 ± 0.062	-0.019 ± 0.081	-0.098 ± 0.057
Trabecular number (1/mm)	0.283 ± 0.077***	0.263 ± 0.061***	0.147 ± 0.088	0.201 ± 0.070**
Trabecular thickness (mm)	-0.073 ± 0.084	-0.099 ± 0.061	-0.010 ± 0.097	-0.095 ± 0.071
Trabecular separation (mm)	-0.229 ± 0.083**	-0.171 ± 0.064**	-0.157 ± 0.094	-0.105 ± 0.073
Trabecular vBMD (mg HA/cm³)	0.192 ± 0.083*	0.146 ± 0.059*	0.130 ± 0.094	0.092 ± 0.065
Trabecular BMC (mg HA)	0.218 ± 0.084**	0.114 ± 0.061	0.196 ± 0.084*	0.033 ± 0.060

*p < 0.05, **p < 0.01, ***p < 0.001. Outcome variables and predictors were standardised to have mean zero and standard deviation of 1.

For Review Only

Table S3. ICE FALCON analyses for the associations of distal radius bone traits adjusted for age and height (outcomes) with lean mass and fat mass (predictors)

**Distal radius bone traits (outcomes)**	Cross-pair cross-trait association		
	β_{cotwin}	s.e.	p
**Lean mass (predictor)**			
Total BMC (mg HA)	0.020	0.064	0.756
Cortical CSA (mm^2)	0.014	0.061	0.820
Trabecular BMC (mg HA)	0.089	0.074	0.230
**Fat mass (predictor)**			
Total vBMD (mg HA/cm^3)	-0.072	0.047	0.126
Cortical vBMD (mg HA/cm^3)	-0.065	0.049	0.184
Trabecular number (1/mm)	0.036	0.066	0.580

Outcome variables and predictors were standardised to have mean zero and standard deviation of 1, p was for 2-side.

ICE FALCON = Inference about Causation through Examination of FAmiliaL CONfounding; s.e. = standard error of the mean; BMC = bone mineral content; HA = hydroxyapatite; CSA = cross-sectional area; vBMD = volumetric bone mineral density.

For Review Only
